# Design and Implementation of a Central-Controllable and Secure Multicast System Based on Universal Identifier Network

**DOI:** 10.3390/s18072135

**Published:** 2018-07-03

**Authors:** Jianfeng Guan, Xuan Liu, Su Yao, Zhongbai Jiang

**Affiliations:** 1 State Key Laboratory of Networking and Switching Technology, Beijing University of Posts and Telecommunications, Beijing 100876, China; liuxua@bupt.edu.cn (X.L.); zbjiang@bupt.edu.cn (Z.J.); 2 Department of Computer Science and Technology, Tsinghua University, Beijing 100084, China; yaosu@tsinghua.edu.cn

**Keywords:** multicast, universal identifier network, SPT, central, controllable, secure

## Abstract

With the rapid increase of network users and services, the breadth and depth of Internet have greatly changed. The mismatch between current network requirements and original network architecture design has spurred the evolution or revolution of Internet to remedy this gap. Lots of research projects on future network architecture have been launched, in which Universal Identifier Network (UIN) architecture that is based on the identifier/location separation, access/core separation and control/forwarding separation can provide better mobility, security and reliability. On the other hand, the demand of group communication has increased due to the fine-grained network services and successive booming of new applications such as IoT (Internet of Things). Most of current multicast schemes are based on the open group model with open group membership (multicast only care the multicast group state, not the group member) and open access to send/receive multicast data, which are beneficial to multicast routing for its simplification. However, the open group membership makes the group member management difficult to be realized, and open access may result in lots of security vulnerabilities such as Denial of service (DoS), eavesdropping and masquerading, which make deployment more difficult. Therefore, in this paper we propose a Central-Controllable and Secure Multicast (CCSM) system based on the UIN architecture, and redesign the multicast service procedures including registration, join/leave, multicast routing construction and update with objective to achieve better mobility support, security, scalability and controllable. More specifically, we design a new group management scheme to perform the multicast members join/leave with authentication and a central-controllable multicast routing scheme to provide a secure way to set up multicast entries on routers. The CCSM inherits the characteristics of UIN in terms of mobility and security, and it can provide the centralized multicast routing computation and distributes the multicast routing into forwarders. We compare CCSM with Protocol Independent Multicast-Sparse Mode (PIM-SM), and the results show that CCSM reduces the multicast join delay, and performs better than PIM-SM in term of reconstruction cost under low multicast density.

## 1. Introduction

The current Internet is derived from ARPANET [1], which has been widespread across the world and permeated into multiple areas. According the recent statistic [2], Internet users in the world had reached about 4.16 billion by the end of 2017. In the same time, the global IP traffic will reach 3.3 ZB by 2021 which is almost three times than 2016 [3]. However, with the booming of Internet, the original architecture design cannot satisfy the current requirements which makes Internet encounter many unprecedented challenges such as poor security, low mobility, and high energy consumption [4]. Besides, the original Internet is designed for a trusted environment with a small number of hosts which are usually from specific organizations and departments. While after 50 years development, with booming of various network technologies, the connotation and denotation of Internet are in the evolution. More specifically, the Internet terminals have shifted from traditional personal computer, notebook, tablet computer and smart-phone to more general things such as various IoT devices, and the Internet services have extended from text-based applications such as web and email to rich media such as live video even Virtual Reality (VR). At the same time, the demand of IP address is also increased greatly which speeds up the transition from IPv4 to IPv6. These shifts are spurring the evolution and the revolution of Internet architecture in terms of mobility, security and scalable. Therefore, lots of future Internet design schemes were proposed in the past several years aiming to alleviate these challenges.

The most recent research has shown that the root causes of current Internet problems are so-called triple bindings, which are resource/location binding, user/network binding and control/data binding [5]. As an representative evolution scheme, Software Defined Network (SDN)/Network Function Virtualization (NFV) is designed to separate the control and forwarding, and decouple the hardware and software, which has been considered as a key technology in 5G core network to provide the programmability [6]. SDN/NFV adopts the softwarization idea to set up network in a software way which is beneficial to network setup, operation, upgrade and management. On the other hand, the revolution schemes such as Information Centric Network (ICN) [7] suggest that Internet should been replaced by clean-state network architecture that takes the information or content as the basic element of network replacing the IP address. ICN aims to decouple the mapping between resource and location, and it introduces the in-network cache to improve the network performance.

Different to SDN and ICN, Universal Identifier Network (UIN) [8,9,10] divides the network protocol stack into pervasive service layer and infrastructure layer, and introduces four identifiers and three mapping mechanisms to decouple the triple bindings. The pervasive service layer consists of virtual service and virtual connection, and it is responsible for session, control and management operations of various services. The virtual service introduces the Service IDentifier (SID) to describe and present various service, and virtual connection provides the various connections identified by Connection IDentifier (CID) for services through the mapping between SID and CID. Based on the SID-CID mapping, UIN decouples the resource/location binding. The infrastructure layer divides the networks into virtual access and virtual backbone. The virtual access is noted as access network, which is designed to handle massive accessing of isomeric users, and adopts the Accessing IDentifier (AID) to identity the various terminals. The virtual backbone is noted as core network, which consists of various network devices and adopts the Routing IDentifier (RID) for routing and data forwarding. Based on this access and core separation mechanism, UIN decouples the user/network binding, and introduces control plane and forwarding plane to decouple the control/data binding. UIN is a promising schemes which has been evolved into Smart Identifier Network (SINET) [11,12] and applied in multiple domains such as vehicular communication [13,14,15], satellite-terrestrial networks [16], wireless sensor networks [17] and smart grid [18].

**Figure 1:**
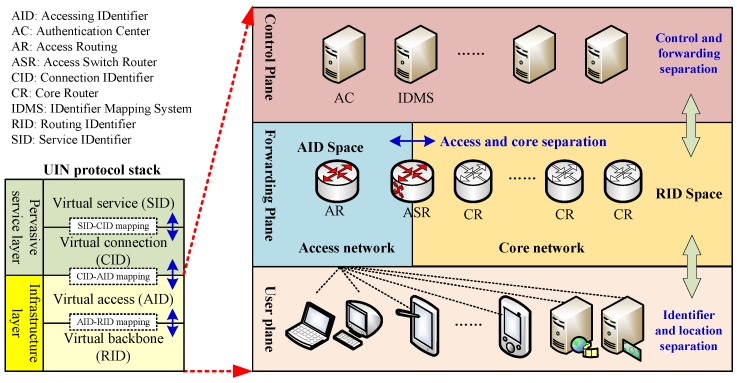
The UIN basic network architecture.

Figure 1 shows the basic network architecture of UIN. As for infrastructure layer, it is composed by three planes including control plane, forwarding plane and user plane. The control plane consists of various control functions, and the basic UIN architecture mainly contains the Authentication Centre (AC) and IDentifier Mapping System (IDMS). AC is responsible for authenticating the attaching users, and IDMS is in charge of the mapping between AID and RID. The forwarding plane consists of access network and core network. The router in core network is called Core Router (CR) and adopts RID as the identifier, while the router in access network is called as Access Router (AR) and uses AID as the identifier. Therefore, there are two addressing spaces in UIN. The user plane consists of various user devices which attach to different access networks. Each device connected to access network will be assigned an AID, and performs the authentication procedure with AC at first. After that, IDMS will assign a AID-RID mapping relationship for authenticated devices. When packets transmit through the network boundary, IDMS will perform the identifier-location mapping management, and Access Switch Router (ASR) will encapsulate RIDs in the head of packets, which would be recognized by router in the core network for forwarding. In this way, massive changes are limited in access networks and the core network will remain relatively stable. The control plane and forwarding plane provide the control and forwarding separation mechanism, while the access network and core network provide the identifier and location separation mechanism. Compared with SDN which only separates the control and forwarding and ICN which only separates the resource and location, UNI provides better mobility, better security and better reliability through the identifier and location separation mechanism, access and core separation mechanism.

The characteristics of UIN are useful for multicast to achieve the better manageable, controllable, and more security [19]. The essence of multicast is to deliver packets from one or more sources to a group of receivers. In general, multicast can be realized on network layer (IP multicast) or application layer. Compared with application layer multicast which provides multicast service based on unicast, IP multicast is designed to save bandwidth consumption of the whole network. The bandwidth benefit that multicast source could gain from IP multicast is huge, mostly from O(*n*) (*n* is the number of destinations in a multicast group) to O(1). After several decades of developments, IP multicast is very popular in specific deployments such as enterprise networks (e.g., for video conferencing), smart home networks (e.g., Universal Plug and Play (UPnP)), carrier IPTV and constrained environments [20]. Most importantly, with the recently fast development of IoTs, massive data from smart objects will be delivered to data centre to abstract the information and vice versa. Therefore, multicast communication plays an important role in this kind of applications.

Currently, the most important IP multicast routing protocols are Protocol Independent Multicast (PIM) and its variants which includes Protocol Independent Multicast-Sparse Mode (PIM-SM) [21], Protocol Independent Multicast-Dense Mode (PIM-DM) [22] and Bidirectional Protocol Independent Multicast (BIDIR-PIM) [23], and all of them have been standardized by IETF. However, these multicast routing protocols are based on the open group model [24,25] and adopt open group management mode through Internet Group Management Protocol (IGMP) [26] and Multicast Listener Discovery (MLD) [27], which cannot support the effective multicast group management for that sources have no control of receivers’ access. For example, the multicast forwarding tree set up by PIM-SM and IGMP/MLD, only knows the multicast states (whether or not the subnet has multicast members), but it lacks of the effective control over multicast members (who joins/leave the multicast group). This operation mode may results in serious security threats such as Denial of service (DoS), eavesdropping and masquerading [28]. In brief, IP multicast has the following problems [19,29,30]:

- IP multicast lacks of an effective multicast source and receiver access control mechanism, and it has no limitations on multicast source and multicast receiver, which makes the deployment of Authentication Authorization Accounting (AAA) service more difficult.
- The membership management in IP multicast is coarse-grained, which takes the router as the basic unit and cannot manage the concrete multicast members.
- The join and leave operation may result in frequently multicast forward tree reconstruction, which have a great impact on the stabilization of multicast services.
- The multicast data lacks of the security mechanism which may result in data leakage.
- IP multicast routing protocol does not record the topology (In resource-constraint environments, maintaining topologies is costly and may not be feasible), and computes the multicast delivery tree in a distributed way, which is difficult to support QoS multicast.

Lots of research efforts aim to improve and enhance multicast controllable, security, and mobility support. However, most of them introduce new complexity, which cannot fundamentally solve these problems. Considering that UIN can provide better mobility and security support, our previous work proposed a new Multicast service model for Identifier/Locator Separation (MILS) mechanism [19] which separates the multicast service identifier and multicast delivery structure to support scalable multicast by different mapping policies. Especially, the MILS introduces the Multicast Controller (MC) to perform the multicast source and receiver authentication, and sets up mapping between access network and core network for multicast to separate the multicast membership management and multicast data delivery. MILS can enhance the multicast security and support multicast mobility. However, it lacks of the detailed operations in term of multicast routing construction. While in this paper, we study the multicast problem based on UIN architecture, and aims to provide controllable and secure multicast scheme. More specifically, we design a Central-Controllable and Secure Multicast (CCSM) based on UIN. We introduce a Multicast Management Center (MMC) as the central controller for multicast service, and multiple MMCs are deployed in order to handle high availability and high concurrency. MMC is responsible for managing the group members and calculates multicast delivery tree. To distribute multicast routing entries, we first calculate the SPTs (Shortest Path Trees) and then send routing information to the relevant routers. The size of core network is the main factor that influences calculation performance. Luckily, the core network is designed to be much smaller than the whole network, so the calculation time is greatly reduced and intensive calculation is feasible.

The contributions of this work can be summarized as follows:

1. We propose a central-controllable and secure multicast based on UIN to provide the centralized multicast membership management and centralized multicast routing mechanism.
2. We design the operation flow of CCSM which includes multicast membership registration, join/leave, multicast tree construction and update, which is a new design different from the current IGMP/MLD and multicast routing protocols.
3. We analyse the mobility, security of CCSM, and evaluate its performance in terms of multicast delivery reconstruction cost and multicast join delay.
4. CCSM adopts the Publish/Subscribe model to provide multicast service which is suitable for IoT applications that deliver the messages among sensors.

The remainder of this paper is organized as follows. Section 2 describes the related work of multicast architecture, multicast security and multicast mobility support. Section 3 describes the system design and main procedure of CCSM. Section 4 evaluates the CCSM and compares it with PIM-SM. Section 5 concludes this paper and summarizes the future directions.

## 2. Related Work

In this section, we investigate the related work of multicast architecture, security and mobility support, and analyse their problems and future development trends.

### 2.1. Multicast Architectures

In this section, we investigate the related multicast architecture under different network architectures, and analyse their characters, and compare them with our proposed scheme.

#### 2.1.1. SDN-Based Multicast

In contrast to traditional IP multicast, SDN-based multicast can enable service providers in a more manageable and flexible manner. Therefore, lots of SDN-based multicast schemes are springing up in recent years [31] with objectives to achieve reliability [32,33], load balance [34], controllability [35] and so on.

Shan-Hsiang Shen et al. [32] proposed a reliable multicast routing for SDN based on recover-aware steiner tree which is a NP-hard problem, and they designed an approximate algorithm called Recover Aware Edge Reduction Algorithm (RAERA) to compute the multicast tree. The introduction of recovery nodes can reduce number of total retransmitted packets and the overall latency that users would experience, which is beneficial for stream providers at the cost of increment on the states in routers. To balance the trade-off between state and bandwidth, Jeremias Blendin et al. [33] proposed an Adaptive Software Defined Multicast (ASDM) scheme which assists ISPs to dynamically adjust the trade-off between bandwidth and state. Their results showed that ASDM can reduce up to 30% bandwidth consumption compared to unicast while only a seventh of network state of multicast is used. Rückert et al. [34] proposed the Dynamic Software-Defined Multicast (DYNSDM) which introduces a network-layer multi-tree approach to distribute traffic and provides the mechanism to support dynamic group and fast reactions. In this way, the DYNSDM can balance the load and accommodate for changing client populations. Besides, to provide multicast member control, Tim Humernbrum et al. [35] proposed a scheme in which multicast source has the full control over group members, and they designed an algorithms for calculating multicast forwarding trees based on Branch-Aware Modification and Early Branching algorithms together to achieve the maximal reusing unicast flow table entries and reduce the utilization of memory.

SDN multicast is based on control and forwarding separation mechanism which is easy to compute the multicast tree in a central way. However, the security and mobility support are not easy to solve.

#### 2.1.2. HIP-Based Multicast

Host Identify Protocol (HIP) is a 3.5 layer protocol which introduces a host identity name space aiming to provide a host layer between network layer and transport layer, and sets up the mapping between host identity and IP to fill the gap between the IP and DNS. By using Host Identity Tag (HIT) as an identifier in transport layer, HIP can provide mobility, multi-homing, and security supports [36]. In this design, host identity represents an abstract concept assigned to a computing platform (end point), and there may be multiple host identities for a given computing platform. While host identifier is a public key which is used as the name of a host identity, and each host identity exactly has one host identifier. HIT is a 128-bit datum created by taking a cryptographic hash over a host identifier plus bits to identify which hash used [37]. HIT is self-certifying and used as the operational representation in the HIP packet header.

The related work and experiences of HIP are described in [38], and some HIP-based multicast schemes were proposed in the past few years. Kovácsházi and Vida [39] proposed a Host Identity Specific Multicast (HISM) model which includes architectural elements, access control, mobility support, and interaction with IP multicast. HISM adopts HIT to replace IP, and introduces HIT-S and HIT-R to represent multicast source and multicast receiver, respectively. Furthermore, HISM proposes a Version Independent Group Management Protocol (VIGMP) to perform group membership management. In term of multicast routing, it inherits the network layer multicast routing protocol such as PIM-SM. Besides, Zhu and Atwood [40] proposed a secure HIP-multicast model by introducing the HIP Multicast Agent (MA) and two-level administrations to provide the authentication/authorization of multicast members and ensure confidentiality of multicast packets. After that, Zhu et al. [41] extended the two-level administrations, and decomposed the HIP-multicast tree into a centre tree and several sub-trees to optimize the multicast delivery of HIP. More recently, Särelä proposed the BloomCasting [42] based on Bloom filter, in which multicast source handles the group management and control the receivers to enable controlled multicast packet forwarding. The HIP-based multicast is still under discuss and neither of them have been adopted by HIP working group, while Bloom filter based scheme may be a promising way for HIP multicast forward [36].

Compared with SDN-based multicast, HIP-based multicast can provide better mobility and security support; however, it lacks of the effective control of multicast tree and the separation mechanism of control and forwarding, which may cause large reconstruction cost when group members frequently join/leave multicast group.

#### 2.1.3. LISP-Based Multicast

Locator/ID Separation Protocol (LISP) [43] is a network layer-based protocol which separates the identification and location of IP address by introducing the Endpoint Identifiers (EIDs) space and Routing Locators (RLOCs) space, respectively. The syntactical format of EIDs and RLOCs is identical to IP address, but their semantics are different. Mapping database is introduced to stores the mapping relationship between EIDs and RLOCs. LISP inherits the traditional host protocol stacks and Internet infrastructure, which is easy to deploy.

Based on LISP framework, LISP multicast [44] is proposed to support the inter-domain multicast routing, which maps the source EID into RLOC without changing the group address, and is compliant with PIM in RLOC name space. Other work such as signal-free LISP multicast [45], is designed to support the multicast packet delivery when multicast is not available to connect the multicast sites together, which adopts unicast replication and encapsulation based on LISP mapping mechanism.

Compared with SDN and HIP-based multicast, LISP-based multicast is easy to deploy due to compliance with IP multicast protocols; however, at the same time it also inherits the shortcomings of IP multicast.

#### 2.1.4. ILNP-Based Multicast

Identifier-Locator Network Protocol (ILNP) [46] is an experimental, evolutionary enhancement to IP, which introduces two distinct name spaces: (1) Identifier which represents a non-topological name for uniquely identifying a node; (2) Locator which represents a topologically bound name for an IP subnet. ILNP uses locator in network layer, and uses Identifier in transport layer, with the objective to split dual roles of IP in terms of identifier and location.

The multicast forwarding and routing in ILNP are unchanged and inherit the IP multicast. Therefore, similar to LISP-based multicast, it inherits the shortcomings of IP multicast.

#### 2.1.5. IoT-Based Multicast

Lots of IoT applications such as service discovery [47], network management and information dissemination [48] will benefit from multicast. Different to the previous multicast routing, IoT multicast cannot maintain the multicast routing topology due to the limited memory and computation capability, which prevents IP multicast being applied to IoT.

To satisfy the requirements of IoT, IETF have published the IPv6 Routing Protocol for Low-Power and Lossy Networks (RPL) [49] which supports built-in multicast by introducing the Modes of Operations (MOPs) 3 to allows messages to carry a multicast address as a destination address. However, RPL has to maintain the topology which is unaccepted for IoT devices, and it also lacks of providing detailed multicast forwarding mechanism. The following research on Multicast protocol for Low-Power and Lossy networks (MPL) [50] aims to provide IPv6 multicast forwarding in constrained networks without constructing or maintaining any multicast forwarding topology. For this purpose, MPL introduces two operation modes. The first mode uses the Trickle algorithm [51] for low density multicast, and the other adopts classic flooding for high density multicast. The most important trait of MPL is the parametrization of Trickle algorithm which can evolve into different dissemination techniques such as flooding and gossip. In this way, MPL can be used in various multicast scenarios and makes the trade-off between latency and efficiency. As an variation of MPL, Stateless Multicast RPL Forwarding algorithm (SMRF) [52] forwards multicast packets in downward direction in the RPL tree. However, SMRF has high end-to-end delay. After that, the Enhanced Stateless Multicast RPL Forwarding (ESMRF) [53] is proposed, which forwards multicast packets up and down the RPL tree. The following work is Bidirectional multicast RPL Forwarding (BMRF) [54], which explores the MOP 3 of RPL and supports bi-directional forwarding and un-subscribe group mechanism. Most recently, a REliable and secure Multicast routing protocol for IoT networks (REMI) [55] is proposed, which supports the cluster creation in RPL tree.

The above multicast schemes aim to provide IPv6 multicast for IoT by considering the constrained network conditions, which inherit the shortcoming of IP multicast. Besides, some application multicast schemes are proposed such as group communication for Constrained Application Protocol (CoAP) [56]; however, application communication loses the advantages of IP multicast in term of bandwidth consumption. Compared with these IoT multicast schemes, our scheme adopts a central control method which separates the multicast routing computation function and multicast data forwarding function from the router, and is easy to realize the multicast routing in resource-limited applications.

From the aspect of multicast architectures, we can find that each multicast architecture has its own emphasis. For example, SDN-based multicast focuses on control capability, and HIP-based multicast focuses on mobility, multi-homing and security support. LISP-based multicast and ILNP-based multicast focus on identifier and location separation, and provide the IP-compatible multicast. IoT-based multicast puts more emphasis on resource-limited. Therefore, in this paper we adopt the UIN as the basic network architecture to study the multicast routing problem, and use the advantages of UIN in terms of mobility, security to provide the secure and controllable multicast services.

### 2.2. Multicast Security Support

The root cause of multicast security problem is derived from open group model [24,25], in which multicast source can send data to any multicast group, and multicast receiver can join or leave the group freely. This model is beneficial in terms of join/leave operation, group member maintenance and multicast routing. However, the open group membership and open access to send/receive multicast data may result in lots of security vulnerabilities such as DoS, eavesdropping and masquerading [28]. IETF Multicast SECurity (MSEC) work group has published an multicast security architecture [57] which contains the multicast data handling, group key management [58] and multicast security policies. Lots of research efforts are focused on multicast data origin authentication [59], multicast receiver and source access control [60], group key distribution [61], establishment [62] and predistribution [63]. Therefore, to insure multicast security, the multicast system should contain multicast source authentication, multicast receiver authentication and multicast data protection.

In our scheme, before attaching to the access network of UIN every host has to perform the authentication via AC, which provides a solid foundation for multicast source and receiver authentication. Besides, with the help of AID-RID mapping between access network and core network, the multicast data can be encrypted easily by ASR.

### 2.3. Multicast Mobility Support

The multicast mobility support generally consists of the mobile source, mobile receiver and mobile forwarder [29], and the root cause of mobile multicast problem is derived from the IP dual properties which means that IP presents the identifier and the location of host. In this case, once mobile multicast source or receiver changes its attachment point, its IP address will be changed. As a result, the multicast session based on IP and port will break, and the multicast service will be disrupted. To solve this problem, lots of research work are based on mobility support protocols such as MIPv6 and PMIPv6 to support mobile multicast [64,65]. However, these mobility support protocols are designed for unicast communication, and direct adoption may introduce additional delivery cost and make mobile multicast inefficient. In fact, the essence of mobility support protocols such as MIPv6 is to separate the identifier and the location properties of IP by introducing the Home of Address (HoA) and Care-of-Address (CoA) to represent the identifier and location of host, respectively. However, to provide the mobility support, all of them are based on fixed anchor such as Home Agent (HA) or Local Mobility Anchor (LMA) to perform the signalling interaction and data forwarding, which may result in the serious single point failure problem. Therefore, the fundamental solution for multicast mobility support is to separate the IP dual properties, and separates mobility signalling management and multicast data forwarding.

In our scheme, the access network and core network adopt the different name spaces which separates the IP dual properties from the network architecture. Therefore, it provides the endogenetic multicast mobility support.

From the above investigations, we can find that multicast has an important potential for applications. However, there is still lack of an effective controlled and secure multicast mechanism. Our previous work have stated the advantages of multicast service in UIN [19], while in this paper, we consider both group management and multicast routing to enhance the mobility and security support.

## 3. System Design

### 3.1. CCSM Basic Components

**Figure 2:**
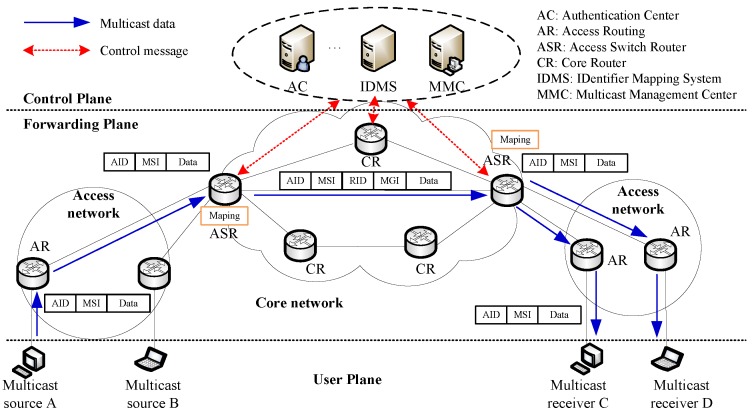
The basic components of CCSM architecture.

To provide the multicast service under UIN, CCSM introduces the Multicast Service Identifier (MSI) and Multicast Group Identifier (MGI) in access network and core network, respectively. MSI is used to identify the logical multicast group relationship which binds to a set of multicast members (In the following section, we use multicast member to represent multicast source and multicast receiver) identified by AIDs. While MGI is used to present a multicast delivery tree which consists of a set of ASR and CR. The introduction of MSI and MGI separates the multicast member management and multicast packet forwarding. The basic components of CCSM are shown in Figure 2, which consists of control plane, forwarding plane and user plane.

The control plane mainly consists of AC, IDMS and MMC. AC is responsible for user access authentication for multicast source and multicast receiver. IDMS is used to manage the mapping between AID and RID, and MSI and MGI. MMC is responsible for multicast membership management (registration, join and leave) and multicast routing construction. The core component of CCSM is MMC which acquires the core network topology, multicast member and related multicast service requirements, and builds the Shortest Path Tree (SPT) as multicast delivery tree for a given multicast group. As for the multicast tree, the root is selected based on the node centrality and the topology is updated in a fixed period so that SPT could always be efficient. We set a root router for a multicast group so that all sources in one group could reuse the SPT. MMC updates the multicast delivery tree in a central way and distributes the multicast routing table into ASR and CR.

The forwarding plane consists of access network and core network. Access network is responsible for user attachment, and it uses AID to represent user device and MSI to represent multicast service, respectively. Core network is responsible for data forwarding which adopts RID to represent the data and MGI to represent multicast service, respectively. The ASR connects access network and core network, and it performs the mapping operations between AID and RID, and MSI and MGI with the assist of IDMS. The user plane consists of multicast sources and multicast receivers, which cooperates with AC to provide the multicast source and receiver authentication.

Based on the CCSM architecture, the multicast membership management is implemented in access network and related group information are stored in MMC for further multicast routing construction. The multicast routing is handled by MMC which computes the multicast delivery tree and installs the multicast routing table in the related ASR and CR. The multicast data delivery between access network and core network performs the mapping and encapsulation procedures to separate the multicast membership management and multicast routing construction. With the help of IDMS, the mapping between MSI and MGI can be one-to-one, one-to-many and many-to-one for different application requirements. Besides, when the access network is multi-hop, the multicast forwarding in access network can base on proxy mechanism like IGMP/MLD proxying [66].

### 3.2. CCSM General Operation Flow

**Figure 3:**
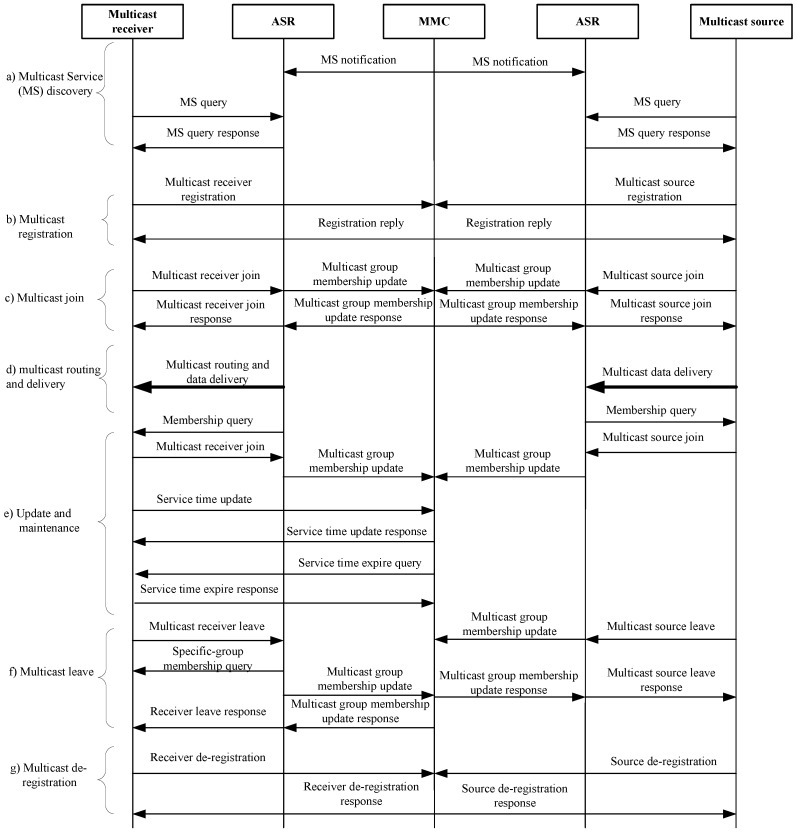
The operation flow of CCSM.

The general operation flow of CCSM is mainly composed by three parts: registration, member join/leave and multicast tree generation procedures. The registration procedure consists of multicast member (multicast source and receiver) registration and de-registration, which performs the multicast service subscription, and assigns a specific authentication code for message authentication, and performs the multicast member access control for security guarantee. The member join/leave procedure performs the join and leave operations which triggers the MMC to perform the multicast delivery tree reconstruction procedure. While the multicast tree generation procedure is responsible for multicast tree construction and maintenance for dynamic changes of multicast members. Besides, CCSM also contains the multicast service discovery procedure to assign the mapping relationship between multicast groups and MMC for large scale development. The detailed operation flow of CCSM is shown in Figure 3, in which multicast receiver is as an example to illustrate multicast member join/leave procedure.

- Multicast service discovery: MMC notifies its multicast service scope, and ASR stores this relation between MMC and its serving MSI scope. In this way, every multicast member will acquire the MMC information for a given MSI.
- Multicast member registration: Multicast member sends source/receiver registration message to MMC to subscribe MSIs. MMC will create the subscription relation between members’ AIDs and their subscribed MSIs. At the same time, MMC will assign authentication codes for members.
- Multicast member join: Multicast member (source and receiver) sends join message to ASR and ASR forwards this join message to update the multicast membership in MMC to update the multicast delivery tree and related multicast routing in ASR/CR.
- Multicast data transmission: Once the multicast routing complete the update, the multicast receiver will get the multicast data, and the multicast source will send the multicast data.
- Update and maintenance: During the multicast service procedure, the multicast membership will be dynamic and therefore the multicast routing has to update in time to avoid the packet loss. The multicast routing update procedure is maintained by MMC based on the up-to-date multicast membership. The multicast routing state update periodically, and at the same time the changes of subscription membership will also trigger the multicast routing update.
- Multicast member leave: Once a multicast member wants to leave certain multicast group, it will send a leave message to MMC, and MMC will perform the multicast routing update procedure to prune the multicast delivery tree. As for a multicast source leave the group, the multicast delivery tree will be suppressed if there are no other sources in that group.
- Multicast member de-registration: Once a multicast member wants to un-subscribe a multicast service, it will send a de-registration message to MMC, and MMC will update the related multicast membership and multicast routing. In this case, the de-registration indicates that the multicast member leaves the group forever.
- Proxy mechanism: This proxy mechanism is similar to IGMP/MLD proxying [66] which can set the proxy node by configuring the upstream interface and downstream interfaces, and maintains a static forwarding structure for multicast members. Notice that, generally there is not routing in access network of UIN for that the AID is used for identifying the host.

### 3.3. Multicast Member Registration Procedure

In CCSM, the multicast source and multicast receiver have to register to MMC at first, and multicast source is responsible for multicast receiver management, and MMC is responsible for multicast service, multicast membership and multicast delivery tree management.

**Figure 4:**
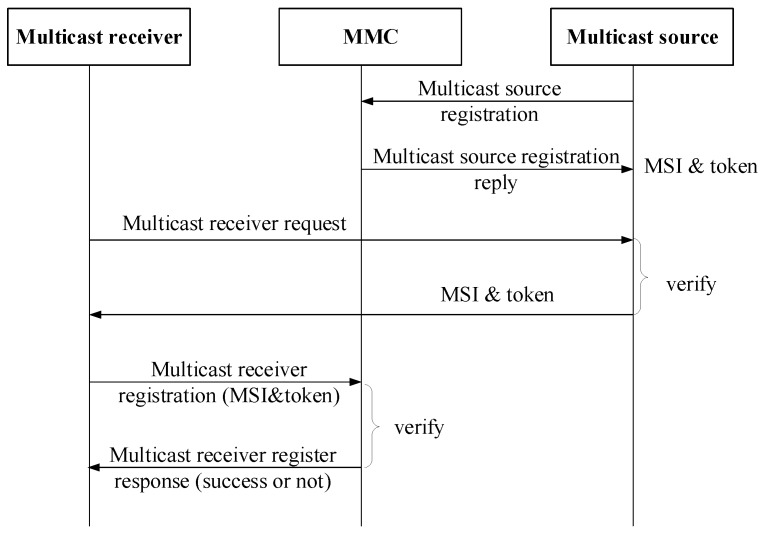
Registration procedure.

If a user in UIN wants to provide a multicast service, it has to register in MMC at first. Then MMC replies to source a multicast address (here called MSI) and a token for secure authentication. If the multicast receivers want to subscribe this service, they have to request source for permission. The source replies to the multicast receivers and transfers the multicast address and tokens. After that, multicast receivers can register to MMC with multicast address and token. MMC maintains the token corresponding to certain multicast address so it can tell whether one multicast receiver is legitimate. Figure 4 shows the registration procedure.

The multicast source in a given multicast group can be multiple. In single-source multicast, the only multicast source executes all the privileges. It registers in MMC, authorizes the receivers and sends multicast packets. While in multi-source multicast, only one multicast source can execute all the privileges, which is called as primary source. The other sources’ privileges are authorized by the primary source.

In this way, CCSM can have full control over the group members and multi-source group could be organized centrally. The token is used in Join/Leave procedure to assure that legitimate users are allowed while illegitimate users will be denied.

### 3.4. Multicast Member Join/Leave Procedure

**Figure 5:**
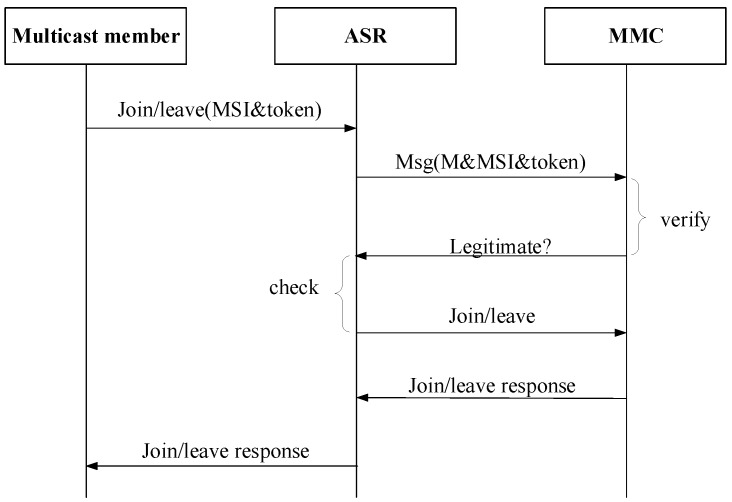
Member join/leave procedure.

Multicast members including source and receiver are all in access networks. When a legitimate receiver wants to join a multicast group, it needs to send a join message with multicast group address (MSI) and matched token to its ASR. Then the ASR transfers this request to MMC to check if this receiver has subscribed this multicast group. If yes, the ASR then checks if there is already other group members in this access network. Only if there is no member joined such multicast group in the access network, ASR will begin to send join message with multicast address, AID and token to MMC. If it has members joined such multicast group, ASR will not send join request to MMC. Figure 5 shows the general member join/leave procedure.

When a multicast source leaves the group, the multicast delivery tree will be suppressed if there are no other sources in this group.

In CCSM, the multicast receiver cannot send join/leave request to MMC directly for that they are located in different name spaces. To forward this join/leave request message, the ASR which multicast receiver attached, has to forward this request for multicast receiver. In this way, MMC maintains the multicast group membership information, and calculates SPT of the core network according to multicast members.

### 3.5. Multicast Tree Generation Procedure

**Figure 6:**
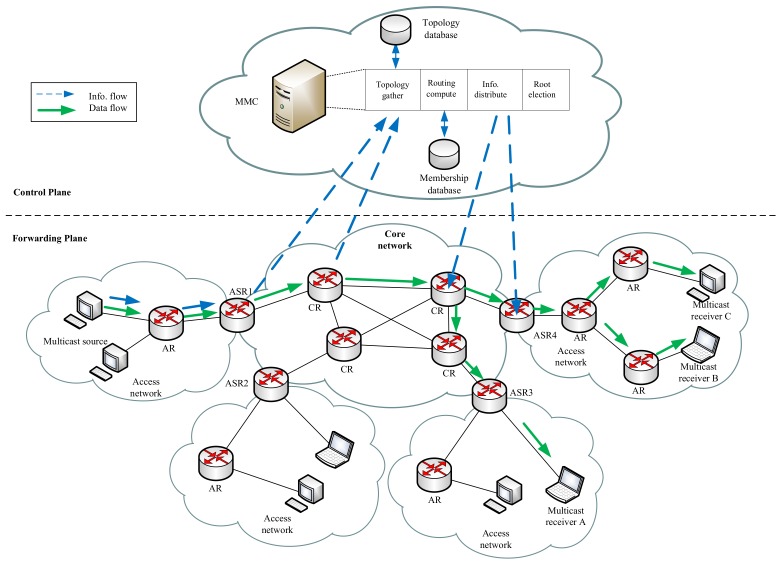
Multicast routing architecture of CCSM.

Figure 6 shows the multicast routing architecture of CCSM which consists of control plane and forwarding plane. Control plane is responsible for gathering network topology, constructing multicast delivery tree, generating and distributing multicast routing information. The core functional entity is MMC which may be multiple. The different MMCs are in charge of different MSIs. Forwarding plane is responsible for packets forwarding in access network and core network, which mainly consists of multicast member, AR, ASR and CR. Once packets traverses the boundary between access network and core network, ASR will perform the mappings of AID-RID and MSI-MGI.

The fundamental of multicast routing is to set up the SPT which represents the forwarding paths of multicast from root to all ASRs that have joined the group. The calculation of SPT is executed in MMC, and needs full core network topology and elected roots as preconditions. In the beginning of CCSM, topology acquiring and roots election need to be done, and they are performed once in a fixed time to ensure the topology and roots election are up to date.

**Figure 7:**
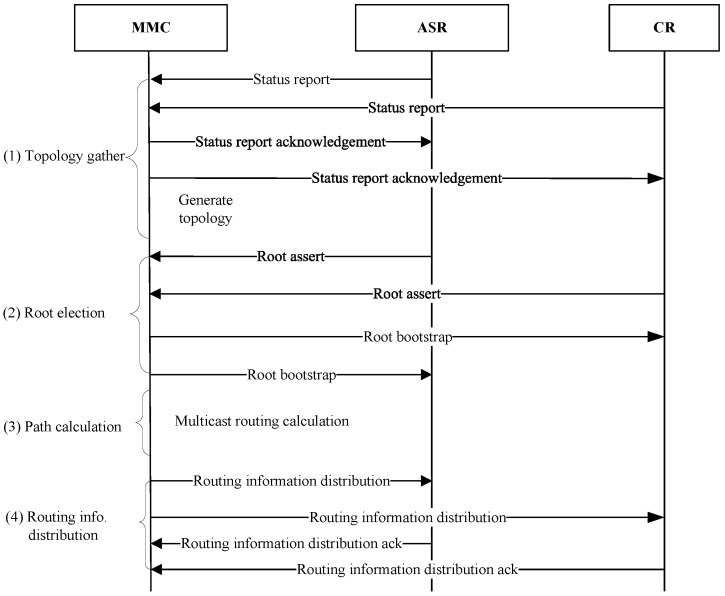
Overview procedure of MMC.

Figure 7 shows the main function flow of MMC which contains four procedure: (1) topology gather; (2) Root election; (3) multicast routing calculation; (4) multicast routing information distribution.

#### 3.5.1. Topology Gather

Topology gather is conducted at the beginning of CCSM. The ASR and CR send the status report message to MMC periodically, and MMC therefore maintains a core network topology. The topology information in MMC have lifetime. Once the lifetime is expired, it will trigger the topology gather procedure. When a new CR/ASR joins in the core network which are infrequent or one CR crashes, acquiring topology is triggered to notify MMC. The topology information consists of device ID, interface ID, relevant interfaces list, and link status information such as bandwidth, delay, jitter and so on.

#### 3.5.2. Root Election

Root election is performed before SPT calculation. During the multicast packets transmission, the source first sends packets to root, and then the root forwards packets along the SPT. When network is small and multicast group is few, one root can satisfy the forwarding requirement. In this case, the root can be designated by MMC and then notifies all the routers in core network. The designation principle should consider the application scenarios and topology characters. For example, MMC can select the root based on node centrality such as degree centrality. When the network is large, there cannot be only one root in the core network for that the traffic is too heavy. In this case, MMC can configure multiple roots for different multicast groups through root bootstrapping mechanism. Besides, CCSM also considers the root update caused by root fault and changes of multicast membership and network topology.

- Root bootstrapping mechanism

The candidate root (ASR or CR) sends the assert message periodically which carries its RID, priority and serving MSI range. MMC collects these assert messages, and adopts the following rules to determine the root for specific MSI, and then generates the mapping between root and MSI, and finally notifies the routers in the core network.

The decision rules are shown as follows:

(1) Compare the priorities of candidate roots, and select the root with highest priority;
(2) If the priority is equal, then compare the hash value according to Equation (1), and select the root with highest hash value;
(3) In the other case, select the candidate root with large RID.

(1) $$Value\left(G,M,C_{i}\right)=\left(1103515245\cdot \left(\left(1103515245\cdot \left(G\&M\right)+12345\right)\;\mathrm{OR}\;C_{i}\right)+12345\right)\;\mathrm{mod}\;2^{31}$$

In Equation (1), Value represents hash value, *G* represents multicast address, *M* represents the hash mask length, $C_{i}$ represents the RID of candidate root, and represents the logic and operator, XOR represents the logical xor operator, and mod represents the modulus operator.

- Root update mechanism

When root is failure, MMC will re-elect root and notifies the other routers, and then triggers the SPT reconstruction. When a specific group membership or network topology is changed, the root does not to be updated.

#### 3.5.3. Path Calculation

When calculating multicast routing and constructing multicast delivery tree, four cases should be considered.

- Case 1: No multicast source, multiple multicast receivers;
- Case 2: One multicast source, multiple multicast receivers;
- Case 3: Multiple multicast sources, multiple multicast receivers;
- Case 4: One or more multicast sources, no multicast receiver.

In case 1, 2, and 3, CCSM will first elects a root and then constructs the SPT, and forwards the multicast packets along the SPT. When a new multicast source joins the multicast group after it finished the multicast source registration procedure, it will first forward the new multicast packets to the root, and then the root will forward the packets to multicast receivers along the SPT. When the packets from the new multicast source arrive the ASR, ASR will look up its multicast routing table. If this access network exists the receivers of that group, ASR will forward the multicast packets to local receivers. For the receivers in this group who are located in other access networks, ASR will forward the multicast packets to root.

In case 4, when multicast source joins a multicast group, the related MMC will find out that there is no multicast receiver in the group, and it will then notify the ASR which multicast source attached. The ASR will discard the multicast packets from the multicast source.

**Figure 8:**
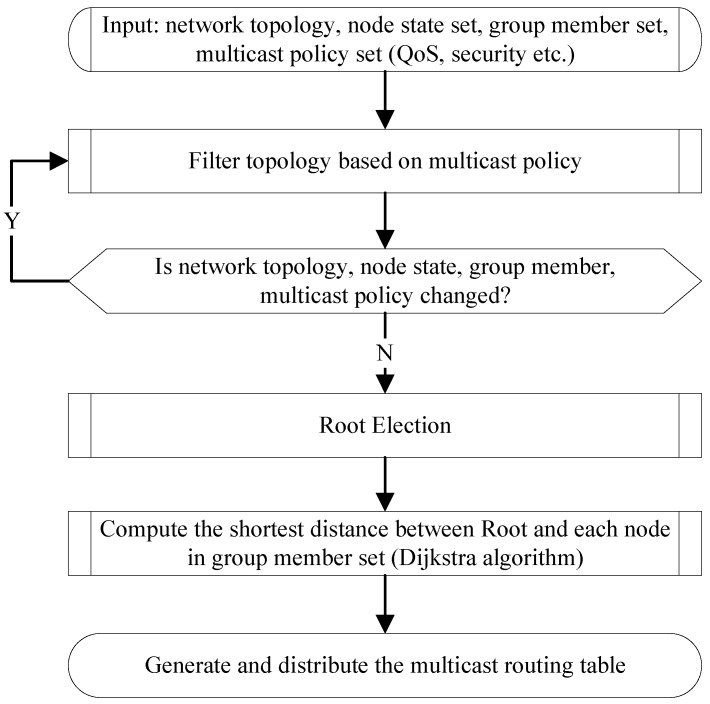
The construction procedure of the path for multicast routing.

The main work of multicast routing is to calculate the SPT. Figure 8 shows the construction procedure of SPT. For a given multicast group, once the root is selected, the SPT from root to each multicast members is easy to calculate. During the calculation, some QoS requirements such as bandwidth/delay can also be considered to satisfy the different application scenarios.

The input information include network topology, node states set, group member set and multicast policy set. Node states record the node overload, resource utilization ratio and so on. Multicast policy such as QoS requirement, and security requirement is used to filter the network topology. The changes of network topology, node state, group member and multicast policy will trigger the filter procedure. Group member set is a set of ASRs which have the multicast receivers. Root election performs the procedure as described in Section 3.5.2. Based on the root and group member set, SPT can be computed based on Dijkstra algorithm [67]. After that, MMC generates and distributes the multicast routing table to ASR/CR on the SPT. In fact, CCSM can select several roots and calculate the SPTs to every ASR and store these SPTs. In this way, CCSM can greatly reduce the time cost when constructing paths for multicast groups.

Once multicast member joins or leaves the group, the SPT will be changed. In this case, MMC has to update the multicast delivery tree. The update procedures are described as follows.

(1) New multicast member join procedure

When a new multicast member joins the group, it will send a join message to ASR to update the membership in the related MMC (notice that every new multicast member should perform the registration and authentication at first to verify its legality). If the ASR has been included in the SPT, the multicast data will delivery to the new member directly. Otherwise, the MMC has to update the multicast delivery tree by calculating a shortest path between this ASR and multicast delivery tree, and distributes the multicast routing information to related CRs.

(2) Multicast member leave procedure

When a multicast member wants to leave the group, it will send a leave message to ASR. ASR will update the membership at first, and then sends a specific-group query to check whether there are other members in this access network. If the access network does not have other members, it will notify the related MMC to update the multicast delivery tree. Otherwise, it updates the multicast routing lifetime.

#### 3.5.4. Multicast Routing Information Distribution

After calculating the up-to-date SPT, MMC will generate the multicast routing entries and distributes them to the related ASR or CR in the SPT. Notice that only MMCs have this authority. Entries installation is very crucial for that too many entries may run out of the routers memory, even cause router paralysis.

### 3.6. Discuss

From the above descriptions, we can summarize that CCSM has the following characteristics.

- CCSM is based on the UIN architecture and inherits the characteristics of UIN in terms of mobility, security and reliability, which means that CCSM can support endogenetic security and mobile multicast.
- CCSM separates the multicast routing computation and multicast data forwarding, which simplifies the multicast routing function on router. Therefore, CCSM is suitable for the scenarios where computing and storage resources are constrained such as IoT applications.
- CCSM provides the multicast member registration mechanism which supports the multicast source and receiver access control, and provides a publish/subscribe model for multicast services.
- CCSM provides the multicast join/leave mechanism which is different from the traditional IGMP/MLD, and it supports the controllable multicast membership management.
- CCSM adopts the centralized multicast routing computation which can easily support various multicast policies including security and QoS, and supports controllable multicast routing, and speeds up the multicast routing convergence.
- CCSM has lots of potential applications due to the existing UIN applications in vehicular communication [13,14,15], satellite-terrestrial networks [16], wireless sensor networks [17] and smart grid [18]. To provide a larger scale deployment of CCSM, some function entities can be implemented in an overlay mode. To be more specific, ASR can be deployed in form of a home router close to the multicast members. The CR can inherit the existing routers. IDMS, AC and MMC can be deployed in the cloud platform.

## 4. Performance Evaluation

In this section, we evaluate CCSM in term of scalability and compare it with the traditional multicast routing protocol. Since the main difference between CCSM and traditional multicast is in the core network, we mainly analyse the amount of multicast entries that CCSM and traditional multicast would install in the core network.

In the evaluation, we choose PIM-SM as a representative of traditional multicast routing. PIM-SM is a distributed multicast routing protocol which cooperates with IGMP/MLD to construct the multicast delivery tree. For a given multicast group, PIM-SM first constructs a shared tree rooted in Rendezvous Point (RP) which is called as RP Tree (RPT). In RPT, multicast source sends multicast packets to RP, and the RP forwards the packets along the RPT, and the multicast entries in router are (*,G). Once multicast receiver gets the multicast packets, it may initiate a transfer from RPT to SPT to optimize latency or bandwidth utilization. Therefore the entries installed in routers will be updated to (S,G). In some implementations, this transfer is based on the load of RP. Unlike PIM-SM, CCSM achieves load balance through dynamic root election in core network, so it is advocated to install (*,G) entries for multicast group without concerning that roots are overloaded. Notice that, the ‘G’ in (*,G) may be MSI or MGI. For multicast group with multiple sources, PIM-SM needs to construct multiple SPTs in line with the number of sources while CCSM only needs to construct one SPT.

We adopt the Inet [68] to generate the network topology and select the root/RP based on node centrality. The network topology size is 4000 and the number of edges is 6659. All the nodes locate in a 10,000 × 10,000 square. For the sake of simplification, we select the node with highest degree centrality as the root, and selects the multicast receiver randomly from the rest of nodes.

In the analysis, we mainly focus on the multicast delivery tree reconstruction cost and multicast receiver join delay. We adopt the hop to measure the reconstruction cost and join delay. The reconstruction cost is measured by the total message delivery distances, and join delay is measured by the distance between multicast delivery tree and new multicast receiver.

**Figure 9:**
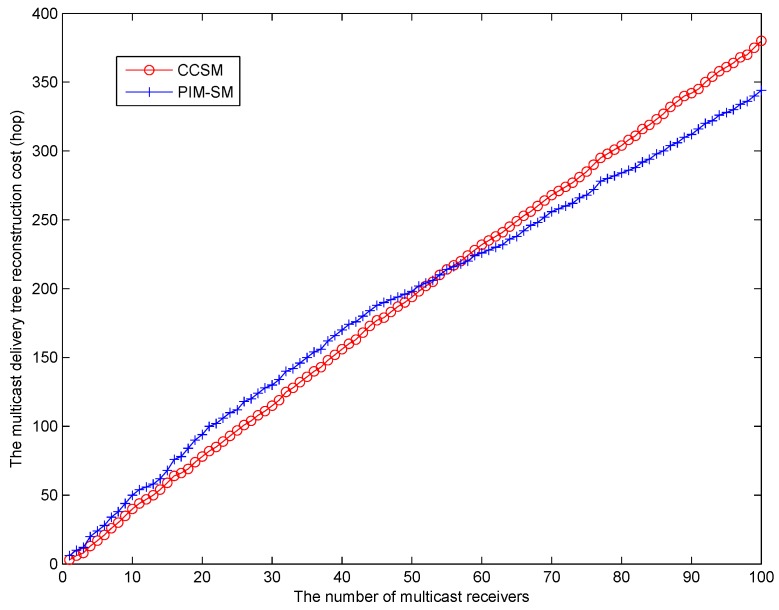
The multicast tree re-construction cost (hop).

Figure 9 shows the reconstruction cost of CCSM and PIM-SM. During this simulation, we randomly select a node as multicast receiver to join the group, and we perform the CCSM multicast reconstruction procedure and PIM-SM graft procedure, respectively. We can find that when the number of multicast receiver is small, to be more specific, less than 54, the reconstruction cost of CCSM is lower than PIM-SM. When the number of receivers is greater than 54, the reconstruction cost of PIM-SM is smaller than CCSM. This is because that with the increase of multicast receivers, the PIM-SM graft procedure will have a high probability to encounter the existing multicast delivery tree, while as for CCSM, all the reconstruction has been initiated by MMC and MMC distributes the multicast routing entries to ASR or CR to update the multicast delivery tree. Therefore, the reconstruction procedure cannot benefit from the existing multicast delivery tree. This result also shows that comparing with PIM-SM, the CCSM is more effective when the multicast receiver density is small.

**Figure 10:**
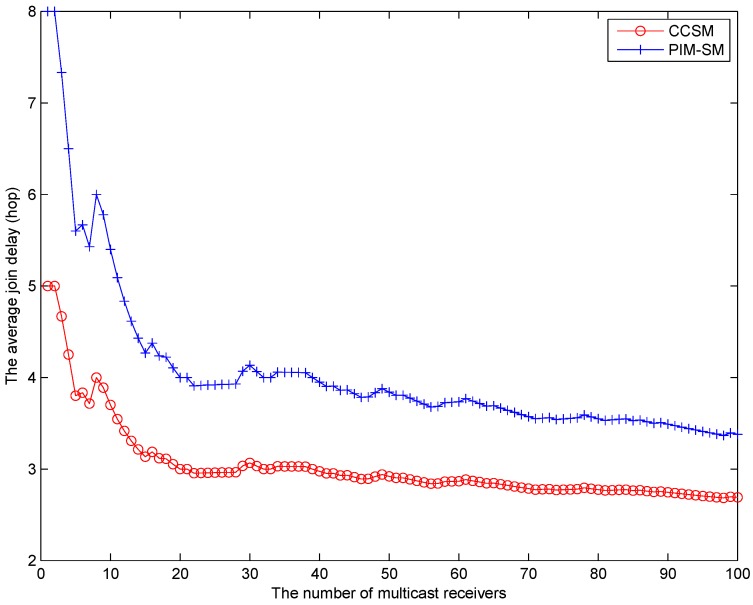
The multicast receiver average join time (hop).

Figure 10 shows the average multicast receiver join delay of CCSM and PIM-SM. We simulate multicast join procedure by randomly selecting a new node as the multicast receiver. When a new multicast receiver joins the group, the multicast delivery tree will be reconstructed. Once the multicast delivery tree finished the update, the new multicast receiver will get the multicast packets. We calculate the average distance of all multicast receivers to get the multicast packets. From Figure 10, we can find that the average join delay of CCSM is lower than PIM-SM although CCSM will take more cost to rebuild the multicast tree when the multicast receiver density is large. The reason is that CCSM adopts the centralized computation method which can set up a global optimal multicast delivery tree for all receivers. On the contrary, PIM-SM adopts the graft method which can only get the local optimal multicast delivery tree. Therefore, the average join delay of CCSM is better than PIM-SM.

## 5. Conclusions

In this paper, we propose a central-controllable and secure multicast system for UIN to overcome the low security and controllability of traditional multicast. The proposed CCSM system demands all members to register in MMC at first and then calculates multicast forwarding paths for legitimate members. This enables MMC to distinguish the legitimacy of member. The root election scheme enables CCSM to reuse one SPT in multi-source groups. Compared with PIM-SM, CCSM has smaller average multicast join delay than PIM-SM, and has lower reconstruction cost than PIM-SM under small multicast receivers density. Besides, based on the inherited character of UIN, CCSM can support endogenetic security and mobile multicast. The design principle of CCSM absorbs the recent research efforts on future network architecture, which is in accordance with the trend of future network. However, there are still some problems that need further study including the source filtering for source-specific multicast and multiple MMCs cooperation.
